# Care of incontinence-associated dermatitis in older adults: A randomized controlled trial comparing zinc oxide–petroleum jelly and centella asiatica–aloe vera preparations

**DOI:** 10.1016/j.ijnsa.2026.100666

**Published:** 2026-08-20

**Authors:** Somphorn Chaisomsee, Samoraphop Banharak, Chakkarin Sommana, Sarunya Tuntiyasawasdikul, Supin Sim-im, Khanisorn Ransinyo, Panita Limpawattana, Waraporn Deesui, Porntip Pimpun, Teerawat Somkamsri, Orada Seeharach

**Affiliations:** a Nursing Department, Srinagarind Hospital, Khon Kaen University, Thailand; b Department of Gerontological Nursing, Faculty of Nursing, Khon Kaen University, Khon Kaen, Thailand; c Department of Pharmacognosy and toxicology, Faculty of Pharmaceutical Sciences, Khon Kaen University, Khon Kaen, Thailand; d Department of Internal Medicine, Faculty of Medicine, Khon Kaen University, Khon Kaen, Thailand; e Department of Internal Medicine, Nampong Hospital, Khon Kaen University, Khon Kaen, Thailand

**Keywords:** Aged, Skin care, Nursing care, Incontinence-associated dermatitis, Incontinence

## Abstract

**Background:**

Incontinence-associated dermatitis is common among older adults in intensive and semi-intensive care units. Impaired consciousness, incontinence, and limited self-care increase the risk of severe skin damage, negatively affecting physical, psychological, and social well-being.

**Aim:**

We compared incontinence-associated dermatitis risk, severity, skin surface pH (a measure of acidity/alkalinity), and skin moisture between the control and experimental groups.

**Design:**

Double blind block-randomized controlled trial, two groups pre-posttest design.

**Methods:**

Participants were randomly assigned to either the control or experimental group. Both participants and outcome assessors were blinded to group allocation. The control group (*n* = 30) received a structured nursing care program for incontinence-associated dermatitis prevention and management combined with zinc oxide mixed with petroleum jelly. The experimental group (*n* = 30) received the same nursing care program combined with an herbal formulation of Centella asiatica and Aloe vera. The intervention lasted 7 days. Outcomes measured included compared incontinence-associated dermatitis risk, severity, skin surface pH, and skin moisture.

**Results:**

The two groups differed significantly (*p* < 0.001), with the experimental group demonstrating significantly lower risk scores than the control group. Similarly, the severity of incontinence-associated dermatitis was significantly lower in the experimental group (*p* < 0.001). Skin pH levels were significantly reduced in the experimental group (*p* < 0.001), indicating a more favorable acidic balance. In addition, skin moisture levels were significantly higher in the experimental group compared to the control group (*p* < 0.001).

**Conclusion:**

A structured nursing care program combined with an herbal formulation of Centella asiatica and Aloe vera effectively reduced the risk and severity of incontinence-associated dermatitis in older adults. Furthermore, the intervention helped maintain optimal skin pH within a mildly acidic range and significantly improved and preserved skin moisture.

**Impact:**

We have provided evidence that a structured nursing care program combined with an herbal-based skin protectant (Centella asiatica and Aloe vera) effectively reduced the risk and severity of incontinence-associated dermatitis in older adults. The intervention improved key skin health indicators, including skin pH balance and moisture, supporting skin integrity and healing. These findings can inform clinical practice by guiding evidence-based incontinence-associated dermatitis prevention and management and by suggesting a cost-effective, accessible approach to improving outcomes in aging populations.

**Patient and Public Contribution:**

Patients and caregivers participated by providing informed consent and supporting intervention implementation. Nursing provided interventions to patients, and a medical doctor conducted the allergic screening and outcome verification.

**Clinical Trial Registration Number:**

TCTR20240910009 (September 10, 2024)

**What is already known**

• Incontinence-associated dermatitis is a common condition among older adults, particularly in semi-intensive and intensive care settings, due to incontinence, immobility, and critical illness
• Incontinence-associated dermatitis increases the risk of complications, such as infection, pressure injuries, prolonged hospitalization, and reduced quality of life in older patients.
• Conventional skin protection products, such as zinc oxide and petroleum-based formulations, are widely used.

**What this paper adds**

• An herbal-based intervention significantly reduced the risk and severity of incontinence-associated dermatitis while improving key skin physiological outcomes, including maintaining a mildly acidic skin pH and increasing skin moisture—both of which are essential for skin barrier function and healing.
• We have highlighted the effectiveness of combining an eight-component nursing care program with herbal products to enhance skin protection and recovery in older patients with incontinence-associated dermatitis.

## 1 Introduction

Incontinence-associated dermatitis is a common skin condition among older adults that adversely affects physical, psychological, familial, and financial well-being (Deprez et al., 2024). It results from prolonged exposure of the skin to urine or feces and is prevalent among older adults due to their high rates of urinary and fecal incontinence (Banharak et al., 2021; Qiao and Banharak, 2023). Hospitalized older adults, particularly those in intensive care units, are at increased risk because of age-related skin changes, acute illness, immobility, cognitive impairment, and incontinence (Deprez et al., 2024; Gunasegaran et al., 2023). In addition to causing pain and discomfort, incontinence-associated dermatitis increases the risk of secondary infections, pressure injuries, prolonged hospitalization, and healthcare costs (Deprez et al., 2024). Although skin barrier products such as zinc oxide and petroleum-based formulations are widely used (Banharak et al., 2021), evidence comparing conventional and herbal-based formulations within structured nursing care programs remains limited. Therefore, further research is needed to support evidence-based nursing practices for preventing and managing incontinence-associated dermatitis in older adults.

## 2 Background

The prevalence of incontinence among hospitalized older adults ranges from 10% to 23% (Leslie et al., 2024). Prolonged exposure of the skin to urine or feces can cause skin inflammation, secondary fungal infections, and pressure injuries, with affected older adults having up to a tenfold greater risk of developing pressure injuries (Banharak et al., 2021; Sommana et al., 2024). Approximately 60% of older adults admitted to semi-intensive care units are at risk of developing incontinence-associated dermatitis (Simim et al., 2024), with incidence rates ranging from 23% to 50% (Coyer and Campbell, 2018). According to the incontinence-associated dermatitis severity categorization tool proposed by the global incontinence-associated dermatitis expert panel (Beeckman et al., 2015), incontinence-associated dermatitis is classified into Category 1 (mild) and Category 2 (moderate to severe). Gunasegaran et al. (2023) reported that 31.8% of participants were in Category 1, while 68.2% were in Category 2. In Thailand, based on the four incontinence-associated dermatitis categories classified by Junkin and Selekof (2008)—early-stage, moderate, severe, and fungal-associated incontinence-associated dermatitis—the reported proportions were 54.8%, 32.3%, 9.7%, and 3.2%, respectively (Simim et al., 2024).

Risk factors for incontinence-associated dermatitis in older adults are multifactorial. Prolonged exposure to urine, feces, and moisture is the primary cause, with skin damage occurring after only 10–15 min of contact (Banharak et al., 2021; Qiao and Banharak, 2023). Diarrhea further increases risk because liquid stool contains digestive enzymes that promote inflammation and excess moisture (Wang et al., 2024). Functional dependence, reduced mobility, and cognitive impairment prolong exposure to irritants and increase skin damage (Deprez et al., 2024; Sommana et al., 2025; Wang et al., 2024). Greater illness severity, including high Acute Physiology and Chronic Health Evaluation II (APACHE II) scores which is a validated severity scoring system used to assess illness severity in critically ill patients, fever, low oxygen saturation, and low Braden scores which is a score from a validated tool used to assess a patient's risk of developing pressure ulcers, is also associated with increased risk (Sommana et al., 2025; Deprez et al., 2024; Coyer and Campbell, 2018). In addition, malnutrition, chronic diseases, and medications such as corticosteroids, diuretics, antibiotics, immunosuppressants, and vasopressors, impair skin integrity, tissue perfusion, and wound healing (Banharak et al., 2021; Coyer and Campbell, 2018; Deprez et al., 2024). Finally, intrinsic skin vulnerability, including higher Perineal Assessment Tool scores and altered skin biomarkers (e.g., pH and moisture), further increases susceptibility to incontinence-associated dermatitis (Simim et al., 2024; Sommana et al., 2024; Chaisomsee et al., 2025).

We conducted a literature review on the prevention and management of incontinence-associated dermatitis, guided by the nursing framework proposed by Banharak et al. (2021). This framework was used to synthesize a systematic approach to care, comprising eight key components: enhancing the knowledge and skills of nursing personnel in incontinence-associated dermatitis prevention; continuous assessment of risk and skin condition; identification of underlying causes and related factors, along with appropriate risk management; proper skin cleansing practices; selection and use of skin protection products; patient positioning to reduce pressure and moisture exposure; nutritional support to maintain skin integrity; and ongoing evaluation of care outcomes. These components reflect a holistic nursing approach that emphasizes comprehensive prevention and management of incontinence-associated dermatitis. Furthermore, Sommana et al. (2024) and Chaisomsee et al. (2025) applied this care framework among older adults admitted to semi-intensive medical units. They reported that the eight care components were effective in protecting and maintaining skin integrity in patients with incontinence-associated dermatitis.

Skin protection products are broadly classified as moisturizers and skin protectants. Moisturizers, including lipid-based occlusive agents and glycerin, maintain skin hydration, whereas skin protectants containing petrolatum, zinc oxide, dimethicone, and acrylic polymers protect the skin from irritation caused by urine and feces (Brennan et al., 2017; Banharak et al., 2021; Glass et al., 2021). However, Thai herbal products remain underutilized in the prevention and management of incontinence-associated dermatitis. Aloe vera promotes wound healing, reduces inflammation, supports tissue repair, and enhances skin hydration (Teplicki et al., 2018; Sharma et al., 2022), while Centella asiatica stimulates collagen synthesis and wound healing (Jenwitheesuk et al., 2018; Witkowska et al., 2024). These complementary properties may improve skin moisture, maintain skin integrity, and facilitate recovery from incontinence-associated dermatitis. Accordingly, we developed a combined herbal-based skin care product containing Aloe vera and Centella asiatica and integrated it into a nursing care program for older adults with incontinence-associated dermatitis. A feasibility study demonstrated its clinical applicability and potential to promote wound healing, improve skin moisture, and maintain skin pH, although confirmation in a larger study was recommended (Chaisomsee et al., 2025).

Sommana et al. (2024) evaluated a nursing care program to prevent incontinence-associated dermatitis in older adults, comparing two intervention groups with usual care. One group received the program with a zinc oxide–petroleum jelly mixture, while the other received zinc oxide followed by petroleum jelly. The latter approach was significantly more effective in preventing incontinence-associated dermatitis, optimizing skin pH, and improving skin moisture (*p* < 0.001). Despite these findings, evidence on the effectiveness of zinc oxide and petroleum jelly specifically for incontinence-associated dermatitis management in older adults remains limited, and Thai herbal-based products are underexplored. Although both conventional agents and herbal products, such as Aloe vera and Centella asiatica, have demonstrated skin-protective properties, direct comparative evidence is lacking—particularly in high-risk populations, such as older adults in semi-intensive medical units. Moreover, previous studies were constrained by small sample sizes and limited evaluation of key nursing outcomes, including risk level, severity, skin pH, and skin moisture. Therefore, we aimed to compare two skin protection approaches—zinc oxide combined with petroleum jelly and Centella asiatica combined with Aloe vera—when integrated with a nursing care program for incontinence-associated dermatitis management, evaluating their effects on risk level, severity, skin pH, and skin moisture among older adults in semi-intensive medical units.

## 3 Aims

We aimed to compare the risk level and severity of incontinence-associated dermatitis, skin pH, and skin moisture between two groups of older adults. The control group received a structured nursing care program combined with a zinc oxide-petroleum jelly skin protectant. In contrast, the intervention group received the same nursing care program combined with an herbal formulation of Centella asiatica and Aloe vera.

## 4 Methods

### 4.1 Design

We employed a double-blinded randomized controlled design. Both participants and outcome assessors were blinded to group allocation to minimize potential bias.

### 4.2 Setting and sampling

#### 4.2.1 Setting

This study was conducted among older patients admitted to a semi-intensive medical care unit in a large university hospital in the northeastern region of Thailand.

#### 4.2.2 Sample size determination

The sample was divided into two independent groups. Sample size estimation was based on a previous study by Jinaporn et al. (2018) conducted in Thailand, which examined the effects of a prevention program for incontinence-associated dermatitis by comparing the control and experimental groups. The parameters used were *P₁* = 0.7857, *P₂* = 0.3571, *α* = 0.05, power (1–*β*) = 0.90, and *Zα* (two-tailed) = 1.96. The sample size was calculated using a formula for comparing two independent proportions (Binu et al., 2014), yielding a sample size of 26 participants per group. To account for a potential dropout rate of 15% (Grove et al., 2013), the final sample size was increased to 30 participants per group, resulting in a total of 60 participants.

#### 4.2.3 Participant selection

4.2.3.1 Inclusion criteria: Patients aged 60 years and older, both male and female, newly admitted to the semi-intensive care unit, either directly from the emergency department or transferred from other units; Patients or legally authorized representatives who were able to provide informed consent; Presence of incontinence with at least three episodes of loose stool within 24 h (Bliss et al., 2017); Diagnosis of incontinence-associated dermatitis.

4.2.3.2 Exclusion criteria: Patients receiving end-of-life palliative care; Patients with known allergies to topical zinc oxide, petroleum jelly, Centella asiatica, or Aloe vera, confirmed by a forearm patch test conducted over 2 h. Participants presenting with itching or rash were excluded.

**Fig. 1:**
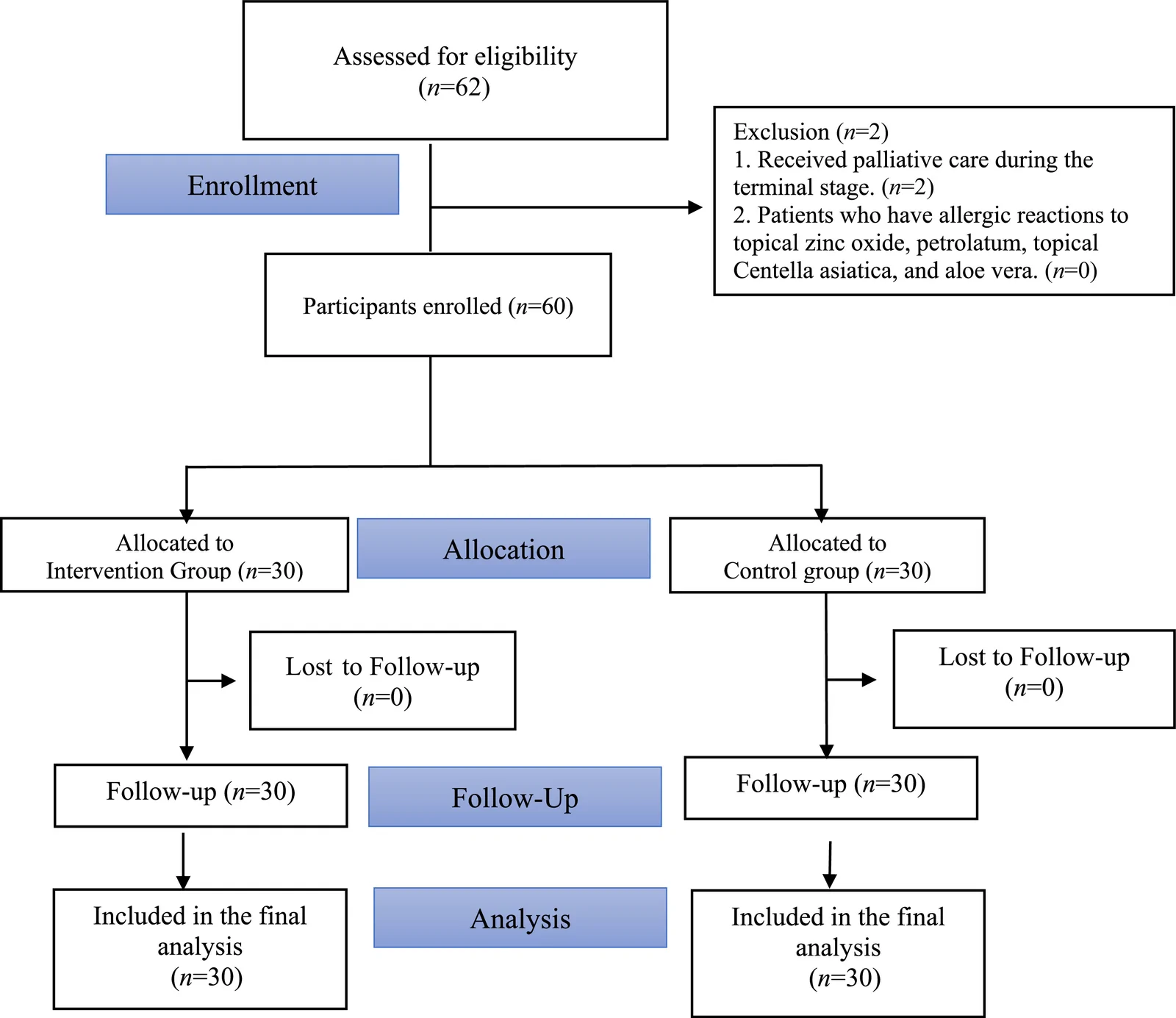
Consort diagram.

4.2.3.3 Withdrawal criteria: Participants who requested to withdraw from the study; Transfer from the unit before completion of the study period; Failure to receive all eight components of the nursing care program daily (Fig. 1).

## 5 Data collection instruments

### 5.1 Screening instruments for participant eligibility

1) The Perineal Assessment Tool (Thai version) (Simim et al., 2024) was used to assess four factors: (1) type, severity, and intensity of irritants; (2) frequency of absorbent product changes; (3) condition of the perineal skin; and (4) contributing factors, including hypoalbuminemia, antibiotic use, enteral feeding, and gastrointestinal infection. Scores were categorized as low risk (4–7 points) and high risk (8–12 points). The tool demonstrated a content validity index (CVI) of 0.90 and an index of item-objective congruence (IOC) of 0.75. Agreement among nurses was 94.5, and a Pearson’s correlation coefficient of 0.94. Inter-rater reliability demonstrated Pearson’s correlation coefficient and Cohen’s kappa values of 0.99 and 0.92, while intra-rater reliability showed values of 0.97 for both coefficients. Older adults with scores below 8 were classified as low risk and were excluded from the study.
2) The Incontinence-Associated Dermatitis Intervention Tool (Simim, 2023) was used to assess incontinence-associated dermatitis severity, including high-risk, early-stage, moderate, severe, and fungal rash. The instrument demonstrated excellent validity, with a CVI and IOC of 1.00. Agreement among nurses was 94.5, and a Pearson’s correlation coefficient was 0.94. Inter-rater reliability demonstrated Pearson’s correlation coefficient and Cohen’s kappa values of 0.96 and 0.94, respectively, while intra-rater reliability showed values of 1.00 for both coefficients. Older adults with incontinence-associated dermatitis who were identified early were considered eligible for study inclusion.
3) Bowel incontinence was assessed by recording at least three episodes of loose stool within 24 h (Bliss et al., 2017). Older adults with fewer than three episodes within 24 h were excluded from the study.

### 5.2 Intervention instruments

The researchers obtained permission to use the nursing care program developed by Sommana et al. (2024), which was based on a systematic review conducted by Banharak et al. (2021). The program’s content validity was evaluated by five experts, including one geriatrician, two wound care specialist nurses, and two gerontological nursing faculty members. The CVI was 1.00, and the IOC was 0.87.

**Fig. 2:**
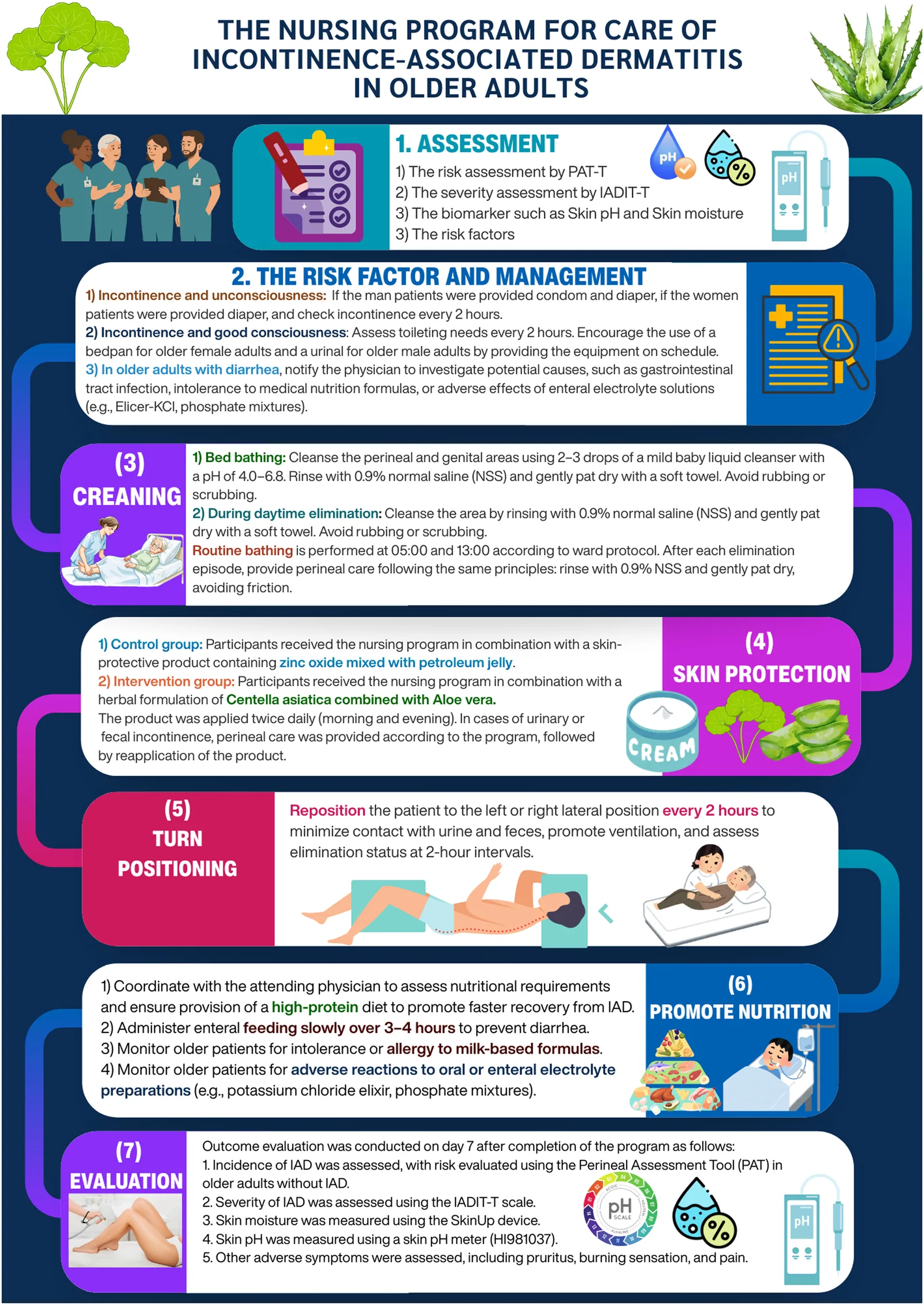
The nursing intervention of care for incontinence-associated dermatitis.

The program consisted of eight nursing care activities for the prevention of incontinence-associated dermatitis: (1) education and skill training care; (2) patient assessment; (3) identification and management of causes and related risk factors; (4) skin cleansing; (5) application of skin protection products, including (a) zinc oxide mixed with petroleum jelly for the control group and (b) a herbal formulation of Centella asiatica combined with Aloe vera for the experimental group. The products were applied twice daily (morning and evening) and reapplied after episodes of urination or defecation following skin cleansing according to the program; (6) repositioning of patients; (7) nutritional support; and (8) outcome evaluation (Fig. 2). Prior to the main study, a pilot study was conducted with 20 participants to assess the program's feasibility. The nursing care program combined with the herbal formulation was acceptable to implement and effective in managing incontinence-associated dermatitis among older adults. Significant improvements were observed in reducing risk and severity levels, maintaining skin pH within a mildly acidic range, and increasing skin moisture. No adverse events or allergic reactions related to the developed product were reported (Chaisomsee et al., 2025).

### 5.3 Data collection instruments

The researchers obtained permission to use the data collection instruments developed by Sommana et al. (2024). The program’s content validity was evaluated by five experts, including one geriatrician, two wound care specialist nurses, and two gerontological nursing faculty members. The CVI was 1.00, and the IOC was 1.00.

#### 5.3.1 Part 1: demographic data form

This section consisted of five items, including general information, sex, age, body mass index, education level, and occupation.

#### 5.3.2 Part 2: clinical data record form

This section included 14 items covering admission diagnosis, comorbidities, APACHE II score, antibiotic use, laxative use, sedative use, level of consciousness, serum albumin level, nutritional status assessed using Society of Parenteral and Enteral Nutrition of Thailand Nutrition Screening Tool (SPENT) and the Nutrition Alert Form, activities of daily living assessed by the Barthel Index for Activities of Daily Living, urinary and fecal continence, use of supportive devices for elimination, use of high-risk medications, and oxygen therapy or mechanical ventilation.

#### 5.3.3 Part 3: outcome assessment form

Outcome measures were assessed at baseline and on day 7th after the intervention by trained research assistants who were blinded to group allocation. The following instruments were used:

1) The Perineal Assessment Tool (Thai version) (Simim et al., 2024), with scores ranging from 4 to 12. Scores of 4–7 indicate low risk, while scores of 8–12 indicate high risk.
2) The Incontinence-Associated Dermatitis Intervention Tool (Supin Simim, 2023), used to classify incontinence-associated dermatitis severity into early-stage, moderate, severe, and fungal rash.
3) Skin moisture was assessed using the SkinUp device (Westermann et al., 2020). The device demonstrated good concurrent validity with the Corneometer, with correlation coefficients ranging from 0.759 to 0.981, despite yielding lower absolute moisture values. Moisture levels were interpreted as follows:

Dry skin (0–39%),

Normal skin moisture (40–60%),

Excessive moisture (61–100%).

4) Skin pH measurement using a Skin pH meter, which measures pH directly on the skin surface. The measurement range is 0.00–12.00, with a resolution of 0.01 and accuracy of ±0.05 (Sommana et al., 2024; Chaisomsee et al., 2025).

A pH value below 7 indicates acidity,

A value of 7 indicates neutrality,

Values above 7 indicate alkalinity.

5) Daily bowel record form, used to document frequency of bowel movements, frequency of changing absorbent products, stool volume, and stool characteristics based on the Bristol Stool Chart.
6) Program adherence checklist, used to monitor the completeness of the eight nursing care activities. Participants were required to receive all components of the program daily to be included in the analysis. Compliance was recorded by the nurses responsible and verified by the head nurse on each shift to ensure accurate implementation.

## 6 Ethical considerations

This study received ethical approval from the Human Research Ethics Committee of XXX University (Approval No HE 661,469, dated October 2, 2024). The study protocol was prospectively registered with the Thai Clinical Trials Registry, under registration number TCTR20240910009 on September 10, 2024. Prior to data collection, participants were provided with comprehensive information about the study's purpose, procedures, data collection duration, and anticipated benefits. Older participants or their legally authorized representatives were informed of their right to voluntarily participate or decline to participate, as well as their right to withdraw from the study at any stage without affecting the care or treatment they received. All data were handled with strict confidentiality, and findings were reported only in aggregate form.

## 7 Validity, reliability and rigor

This study was conducted in strict accordance with the research protocol. In terms of study design, rigorous methodological procedures were applied, including block randomization with allocation concealment using sealed envelopes. A double-blind approach was implemented, with both participants and outcome assessors unaware of group allocation to minimize potential bias. Prior to data collection, registered nurses (RNs) and nursing assistants were trained in implementing the intervention to ensure consistent and accurate delivery of the program. Trained research assistants were assigned to conduct baseline and outcome assessments, and they were blinded to group assignment to reduce measurement bias. Regarding data collection procedures, a clear operational plan was established. A structured checklist was used to monitor daily adherence to all eight nursing care activities, ensuring the intervention was implemented according to the protocol. The instruments and intervention program used in this study were based on standardized tools previously applied in related research. Outcome assessments were conducted systematically at both pre- and post-intervention stages, and data completeness was verified prior to analysis.

## 8 Data collection procedures

After obtaining ethical approval and registering the study, formal permission was requested from hospital administrators and unit managers to conduct data collection. Meetings were held with ward leaders to explain the research procedures. In addition, training sessions were provided for RNs and nursing assistants involved in the study to ensure accurate and consistent implementation of the intervention. The research team also conducted meetings to clarify data collection procedures and develop a master plan to guide the study process, as follows:

1) Research assistants were responsible for outcome assessment. They were trained to use all assessment tools, including skin assessment devices. The research assistants who performed baseline and post-intervention assessments were blinded to group allocation and conducted evaluations upon notification by the principal investigator after each participant completed the intervention.
2) The principal investigator screened participants according to the inclusion and exclusion criteria. Eligible participants were then randomly assigned using block randomization with allocation concealment through sealed envelopes. Participants were not aware of their group assignment.
3) After enrollment was completed, RNs and nursing assistants providing care were blinded to the type of skin protection product used. The products were distinguished only by different-colored labels on the bottom of the containers. Both groups received the same eight-component nursing care program for incontinence-associated dermatitis over 7 days.
4) The head nurse or shift leader supervised and monitored the implementation of the program using a structured checklist. Completion of all eight nursing care activities was recorded daily by the nurses responsible and verified by the shift leader to ensure adherence to the protocol.

## 9 Data analysis

Descriptive statistics were used to summarize participants’ characteristics, including percentages, means, standard deviations, medians, minimum values, and maximum values. Differences in categorical variables were examined using the chi-square test or Fisher’s exact test, as appropriate. For continuous variables, comparisons were conducted using the independent *t*-test or the Mann–Whitney U test. The continuous outcome variables included risk scores, severity levels, skin pH, and skin moisture. Data distribution was assessed using the Shapiro–Wilk test, which indicated non-normal distribution (*p* < 0.001). Therefore, the Wilcoxon signed-rank test was used for within-group comparisons, and the Mann–Whitney U test was applied for between-group comparisons.

## 10 Results

### 10.1 Demographic information

The majority of participants were female, with the largest proportion in the 70–79-year age group. Regarding pre-retirement occupation, most participants had been engaged in agriculture (Table 1).

**Table 1:** Demographic characteristics and history of illness and risk factor among older patients (*N* = 60).

Demographic characteristics	Intervention *n* (%)	Control *n* (%)	All *n* (%)	*p*-value
Sex				0.796^c^
Male	15 (50.00)	16 (53.30)	31 (51.70)	
Female	15 (50.00)	14 (46.70)	29 (48.30)	
Age (years)				0.183^F^
60–69 years	12 (40.00)	5 (16.70)	17 (28.30)	
70–79 years	11 (36.70)	15 (50.00)	26 (43.30)	
80 years up	7 (23.30)	10 (33.30)	17 (28.30)	
(Min–Max)	(61–96)	(60–92)	(60–96)	0.164^I^
(Mean ±S.D.)	(74.40 ± 9.95)	(76.67 ± 8.04)	(75.53 ± 9.04)	
Body mass index (kg / m^2^)				0.109^F^
Less than 18.50	5 (16.70)	9 (30.00)	14 (23.30)	
18.50–24.99	13 (43.30)	16 (53.30)	29 (48.30)	
more than 24.00	12 (40.00)	5 (16.70)	17 (28.30)	
(Min–Max)	(13.15–37.95)	(12.07–26.67)	(12.07–37.95)	
(x¯ ±S.D.)	(23.20 ± 5.53)	(20.66 ± 4.09)	(21.93 ± 4.99)	
Highest education level				0.656^F^
Uneducated	15 (50.00)	14 (46.70)	29 (48.30)	
Primary school	7 (23.30)	6 (20.00)	13 (21.70)	
secondary school	2 (6.70)	4 (13.30)	6 (10.00)	
Bachelor’s degree	4 (13.30)	6 (20.00)	10 (16.70)	
Master’s degree up	2 (6.70)	0	2 (3.30)	
Occupation				0.511^F^
Unemployed	7 (23.30)	7 (23.30)	14 (23.30)	
Agriculturist	11 (36.70)	8 (26.70)	19 (31.70)	
Government	8 (26.70)	6 (20.00)	14 (23.30)	
Seller/ business owner	3 (10.00)	4 (13.30)	7 (11.70)	
Others	1 (3.30)	5 (16.70)	6 (10.00)	
Cause of admission (More than 1 answer can be given)				
Respiratory disease	23 (76.70)	21 (70.00)	44 (73.30)	0.559^C^
Cardiovascular disease	3 (10.00)	4 (13.30)	7 (11.70)	0.500^F^
Congestive heart failure	2 (6.70)	6 (20.00)	8 (13.30)	0.254^F^
Chronic kidney disease	2 (6.70)	3 (10.00)	5 (8.30)	0.500^F^
Septic shock	19 (63.30)	19 (63.30)	38 (63.30)	0.605^C^
Infection	21 (70.00)	18 (60.00)	39 (65.00)	0.417^C^
Others	8 (26.70)	11 (36.70)	19 (31.70)	0.405^C^
Chronic diseases (More than 1 answer can be given)				
Hypertension	15 (50.00)	14 (46.70)	29 (48.30)	0.796^C^
Diabetes	16 (53.30)	13 (43.30)	29 (48.30)	0.438^C^
Hyperlipidemia	16 (53.30)	8 (26.70)	24 (40.00)	0.035^C^
Cardiovascular disease	9 (30.00)	7 (23.30)	16 (26.70)	0.559^C^
Gout	5 (16.70)	3 (10.00)	8 (13.30)	0.706^F^
BPH	2 (6.70)	4 (13.30)	6 (10.00)	0.671^F^
COPD	8 (26.70)	8 (26.70)	16 (26.70)	1.000^C^
Dementia	5 (16.70)	7 (23.30)	12 (20.00)	0.519^C^
Stroke	9 (30.00)	8 (26.70)	17 (28.30)	0.774^C^
Hepatitis	5 (16.70)	1 (3.30)	6 (10.00)	0.195^C^
Anemia	4 (13.30)	3 (10.00)	7 (11.70)	1.000^C^
Chronic kidney disease	5 (16.70)	5 (16.70)	10 (16.70)	1.000^C^
Others	9 (30.00)	3 (10.00)	12 (20.00)	0.053^C^
APACHEII score				
(Min–Max)	(10–31)	(9–36)	(9–36)	0.518^I^
(x¯ ±S.D.)	(22.37 ± 5.11)	(22.20 ± 5.85)	(22.28 ± 5.44)	

Demographic characteristics and history of illness and risk factor among older patients (Cont.).				
Demographic Characteristics	Intervention *n* (%)	Control *n* (%)	All *n* (%)	*p*-value
Serum albumin level (g/dL)				
(Min–Max)	(1.70–4.60)	(1.10–3.20)	(1.10–4.60)	0.518^M^
(x¯ ±S.D.)	(2.62 ± 0.53)	(2.29 ± 0.42)	(2.55 ± 0.48)	
Consciousness (GCS)				
(Min–Max)	(6–15)	(3–15)	(3–15)	0.697^M^
(x¯ ±S.D.)	(10.83 ± 2.17)	(11.00 ± 2.60)	(10.92 ± 2.37)	
Level of Consciousness				0.913^F^
Mild (13–15)	3 (10.00)	0	8 (13.30)	
Moderate (9 - 12 score)	23 (76.70)	26 (86.70)	44 (73.30)	
Severe (≤ 8 score)	4 (13.30)	4 (13.30)	8 (13.30)	
Used of sedative drugs				0.647^F^
No	26 (86.70)	26 (86.70)	52 (86.70)	
Yes	4 (13.30)	4 (13.30)	8 (13.30)	
Used of laxative drugs				1.000^C^
Yes	12 (40.00)	12 (40.00)	24 (40.00)	
No	18 (60.00)	18 (60.00)	36 (60.00)	
Type of laxative drugs				0.912^F^
Senokot	2 (6.70)	3 (10.00)	5 (8.30)	
Lactulose	8 (26.70)	8 (26.70)	16 (26.70)	
Senokot and Lactulose	2 (6.70)	3 (3.30)	3 (5.00)	
Used of antibiotic drugs				0.492^F^
Yes	30 (100)	28 (93.30)	58 (96.70)	
No	0	2 (6.70)	2 (3.30)	
Number of antibiotic drugs				0.363^F^
1 type	5 (16.70)	9 (32.14)	14 (23.30)	
2 types	20 (66.70)	14 (50.00)	34 (56.67)	
3 types	4 (13.30)	4 (14.29)	8 (13.33)	
4 types up	1 (3.30)	1 (3.57)	2 (3.30)	
Barthel Activities of Daily Living				
(Min–Max)	(0–7)	(0–13)	(0–13)	0.962^M^
(x¯ ±S.D.)	(1.07 ± 2.20)	(1.37 ± 3.01)	(1.22 ± 2.62)	
Risk of malnutrition (SPENT)				
High risk	30 (100.00)	30 (100.00)	60 (100.00)	1.000^C^
Nutrition status (NAF)				0.488^C^
Moderate malnutrition	4 (13.30)	6 (20.00)	10 (16.70)	
Severe malnutrition	26 (86.70)	24 (80.00)	50 (83.30)	
(Min–Max)	(9–21)	(9–23)	(9–23)	0.889^M^
(x¯ ±S.D.)	(15.97 ± 3.25)	(16.00 ± 3.71)	(15.98 ± 3.46)	
Used of the Oxygen therapies				
Yes	28 (93.30)	27 (90.00)	55 (91.70)	0.500^F^
No	2 (6.70)	3 (10.00)	5 (8.30)	
Type of the Oxygen therapies				0.959^F^
Cannula	0	1 (3.30)	1 (1.70)	
Mask with bag	0	1 (3.30)	1 (1.70)	
HFNC	4 (13.30)	4 (13.30)	8 (13.30)	
ET-Tube	17 (56.70)	15 (50.00)	32 (53.30)	
TT-Tube	8 (26.70)	7 (23.30)	15 (25.00)	

Demographic characteristics and history of illness and risk factor among older patients (Cont.).				
Demographic Characteristics	Intervention *n* (%)	Control *n* (%)	All *n* (%)	*p*-value
Used of Vasopressors				0.194^F^
Yes	16 (53.30)	11 (36.70)	27 (45.00)	
No	14 (46.70)	19 (63.30)	33 (55.00)	
Type of Vasopressors				0.194^C^
Norepinephrine	16 (53.30)	11 (36.70)	27 (45.00)	
Restrained				0.774^C^
Yes	21 (70.00)	22 (73.30)	43 (71.70)	
No	9 (30.00)	8 (26.70)	17 (28.30)	
Incontinence Management (More than 1 answer can be given)				
Diaper	30 (100.00)	30 (100.00)	60 (100.00)	1.000^C^
Retrained Foley catheter	15 (50.00)	13 (43.30)	28 (46.70)	0.605^C^
Condom	3 (10.00)	6 (20.00)	9 (15.00)	0.472^F^
Stool examination				0.152^C^
Yes	24 (80.00)	19 (63.30)	43 (71.70)	
No	6 (20.00)	11 (36.70)	17 (28.30)	
Result of Stool examination				0.451^F^
Non infection	22 (73.30)	25 (83.33)	47 (78.30)	
Infection	8 (26.70)	5 (16.67)	13 (21.70)	
Bristol Stool Chart				
(Min–Max)	(5–7)	(6–7)	(5–7)	0.312^M^
(Mean ±S.D.)	(6.73 ± 0.52)	(6.63 ± 0.49)	(6.68 ± 0.50)	
Change diaper per day				
(Min–Max)	(3–8)	(4–7)	(3–8)	0.377^M^
($\bar{x}$ ±S.D.)	(5.53 ± 1.22)	(5.30 ± 0.88)	(5.42 ± 1.06)	
Oral potassium (Elixir KCl)-related diarrhea			0.037^C^	
Yes	4 (13.30)	11 (36.70)	15 (25.00)	
No	26 (86.70)	19 (63.30)	45 (75.00)	
Clinical Consequences of IAD; e.g., pain			0.096^F^	
Yes	22 (73.30)	28 (93.30)	50 (83.30)	
No	8 (26.70)	2 (6.70)	10 (16.70)	

Note: Note: C=Chi-square; F=Fisher’s exact test; M=Mann Whitney U test; I=Independence *t*-test; Min=minimum; Max=maximum; x¯ = mean; S.D.=standard deviation; IAD= incontinence-associated dermatitis; B**PH – benign prostatic hyperplasia; COPD=**chronic obstructive pulmonary disease; APACHE II = acute physiology and chronic health evaluation II.; GCS= glasgow coma scale.; SPENT nutrition screening tool = society of parenteral and enteral nutrition of Thailand nutrition screening tool; NAF= nutrition alert form.; HFNC= high flow nasal cannula.; ET-Tube= endotracheal tube.; TT-Tube= tracheostomy tube.

### 10.2 History of illness and risk factor

Most participants were admitted with respiratory diseases, followed by systemic infections. In terms of comorbidities, hypertension and diabetes mellitus were equally prevalent. Disease severity level was high.

Regarding risk factors for incontinence-associated dermatitis, no significant differences were observed between the two groups. Most participants had a moderate level of consciousness, did not receive sedative medications, used lactulose as the primary laxative, and received multiple antibiotics, typically two.

Most participants had a high level of dependency and were at high nutritional risk, with the majority classified as having severe nutritional status. Most received oxygen therapy via an endotracheal tube; nearly half received vasopressors (primarily norepinephrine); most had an indwelling urinary catheter; and all used absorbent products (Table 1).

### 10.3 The perineal assessment tool

Within-group comparisons showed significant improvements in both the control and experimental groups following the intervention. Between-group comparisons indicated no baseline differences; however, after the intervention, the experimental group demonstrated significantly lower risk scores than the control group (Table 3).

### 10.4 The severity of incontinence-associated dermatitis

Within-group comparisons showed significant reductions in the severity of incontinence-associated dermatitis in both the control and experimental groups following the intervention. There were no baseline differences between the groups; however, the experimental group demonstrated significantly lower severity after the intervention and a greater reduction in severity than the control group (Table 3).

### 10.5 Skin pH

Within-group comparisons showed significant changes in skin pH in both the control and experimental groups following the intervention. There were no baseline differences between the groups; however, after the intervention, the experimental group had significantly lower skin pH than the control group (Table 2).

**Table 2:** The comparison risk and biomarkers of IAD before and after receiving interventions of within group comparison and between groups comparison among older patients (*N* = 60).

within group comparison												
Outcomes	Groups	Before		After		Mean rank	Sum of Rank	Z^W^	*p*-value			
		x¯	SD	x¯	SD							
PAT-T score	Intervention	10.23	0.94	6.40	0.89	15.50	465.00	−4.982	<0.001			
	Control	9.90	0.85	8.13	1.68	8.75	17.50	−3.942	<0.001			
Skin surface pH	Intervention	7.51	0.34	6.07	0.32	15.50	465.00	−4.782	<0.001			
	Control	7.43	0.43	7.07	0.55	10.70	107.00	−2.582	0.010			
Skin moisture	Intervention	10.20	2.83	13.45	1.12	15.50	465.00	−4.784	<0.001			
	Control	10.22	0.28	11.41	1.46	16.66	416.50	−3.791	<0.001			

between groups comparison												
Measured time	Outcome	Control				Intervention				Mann-Whitney U Test	Z^M^	*p* -value
		x¯	S.D.	Mean rank	Sum of Ranks	x¯	S.D.	Mean rank	Sum of Ranks			
Before	PAT-T score	9.90	0.85	27.13	814.00	10.23	0.94	33.87	1016.00	349.00	−1.598	0.110
	Skin surface pH	7.43	0.43	29.10	873.00	7.51	0.34	31.90	957.00	408.00	−0.621	0.535
	Skin moisture	10.22	0.28	29.52	885.50	10.20	2.83	31.48	994.50	420.50	−0.442	0.658
After	PAT-T score	8.13	1.68	39.20	1176.00	6.40	0.89	21.80	654.00	189.00	−3.992	<0.001
	Skin surface pH	7.07	0.55	43.70	1311.00	6.07	0.32	17.30	519.00	54.00	−5.856	<0.001
	Skin moisture	11.41	1.46	19.18	575.50	13.45	1.12	41.82	1254.50	110.50	−5.027	<0.001

Note: IAD= incontinence-associated dermatitis; x¯ = Mean; PAT-T= Perineal Assessment Tool Thai version; S.D. = Standard Deviation; Z^W^=Wilcoxon signed rank test; Z^M^= Mann-Whitney U Test.

**Table 3:** The comparison of the severity of IAD of within groups comparison and within groups comparison among older patients (*N* = 60).

within groups comparison																
Groups	Time	High risk *n* (%)	Mild *n* (%)	Moderate *n* (%)	Severe *n* (%)	Mean rank	Sum of rank	Z^W^	*p*-value							
Control	Before	0	7 (23.30)	12 (40.00)	11 (36.70)	11.47	195.00	−2.954	0.003							
	After	3 (10.00)	14 (46.70)	6 (20.00)	7 (23.30)											
Intervention	Before	0	2 (6.70)	2 (63.30)	9 (30.00)	15.50	465.00	−4.950	<0.001							
	After	17 (65.70)	12 (40.00)	1 (3.30)	0											

between groups comparison																
Measured time	Outcome	Control						Intervention						Mann-Whitney U Test	Z^M^	*p*-value
		High risk *n* (%)	Mild *n* (%)	Moderate *n* (%)	Severe *n* (%)	Mean rank	Sum of Ranks	High risk *n* (%)	Mild *n* (%)	Moderate *n* (%)	Severe *n* (%)	Mean rank	Sum of Ranks			
Before	Severity	0	7 (23.30)	12 (40.00)	11 (36.70)	29.68	980.50	0	2 (6.70)	2 (63.30)	9 (30.00)	39.98	1199.50	425.50	−0.400	0.689
After	Severity	3 (10.00)	14 (46.70)	6 (20.00)	7 (23.30)	31.32	939.50	17 (65.70)	12 (40.00)	1 (3.30)	0	21.02	630.50	165.50	−4.487	<0.001

The difference comparison of the severity of IAD after intervention between groups comparison																
Outcome	Control						Intervention						Mann-Whitney U Test	Z	*p*-value	
	Better *n* (%)	Good *n* (%)	Same *n* (%)	Bad *n* (%)	Mean rank	Sum of Ranks	Better *n* (%)	Good *n* (%)	Same *n* (%)	Bad *n* (%)	Mean rank	Sum of Ranks				
Δ Severity	4 (13.30)	13 (43.30)	9 (30.00)	4 (13.30)	41.08	1232.50	21 (70.00)	9 (30.00)	0	0	19.92	597.50	132.50	−4.973	<0.001	

Note: IAD= incontinence-associated dermatitis.; Z^W^=Wilcoxon signed rank test; Z^M^= Mann-Whitney U Test.; Δ= difference of severity.

### 10.6 Skin moisture

Within-group comparisons showed significant changes in skin moisture levels in both the control and experimental groups following the intervention. There were no baseline differences between the groups; however, after the intervention, the experimental group demonstrated higher skin moisture levels than the control group (Table 2).

## 11 Discussion

The demographics are consistent with previous studies by Sommana et al. (2024); Glass et al. (2021), and Sommana et al. (2025). Incontinence-associated dermatitis appears to occur more frequently in female patients, particularly among those admitted to intensive care settings who are unable to control elimination or communicate their needs effectively. Female patients with incontinence often require the use of absorbent products, which increases the likelihood of prolonged skin exposure to urine or feces in the perineal area. Repeated exposure to moisture and irritants can compromise skin integrity and contribute to the development of incontinence-associated dermatitis. In contrast, the lower incidence observed in male patients may be explained by the use of external urinary devices, such as condom catheters, which help prevent direct contact between urine and the skin. As a result, the risk of skin damage and subsequent incontinence-associated dermatitis may be reduced in male patients compared with female patients.

Using the nutrition screening tool indicated that all participants were at high risk, and most had severe nutritional status based on the nutrition alert form, supported by low serum albumin levels. Malnutrition may impair skin moisture tolerance, barrier function, tissue repair, and inflammatory responses (Gray and Giuliano, 2018). In older adults with hypoalbuminemia, exposure to urine and feces containing alkaline components and digestive enzymes may accelerate skin damage (Ruiz-Rosso et al., 2024). In addition, inadequate protein intake impairs epidermal regeneration, collagen synthesis, and skin barrier restoration, increasing skin fragility and susceptibility to irritation (Banharak et al., 2021; Jiang et al., 2025; Ruiz-Rosso et al., 2024). Therefore, malnourished older adults with severe illness or dependency are at particularly high risk of developing incontinence-associated dermatitis.

Most participants experienced severe illness, reflecting the critical condition of older adults with incontinence-associated dermatitis admitted to semi-intensive or intensive care units, where limited mobility and high dependency are common (Sommana et al., 2025). These conditions increase the risk of complications, particularly incontinence-associated dermatitis and pressure injuries (Chang, 2024; Wang et al., 2024). Respiratory impairment was common, with many participants requiring endotracheal intubation. Hypoxia may impair cellular energy production, disrupt skin barrier function, increase transepidermal water loss, and reduce skin moisture, thereby increasing susceptibility to irritation (Wang et al., 2024). Additionally, reduced perfusion in critically ill older adults often requires vasopressor support, such as norepinephrine, which induces peripheral vasoconstriction and may decrease skin blood flow (McEvoy et al., 2022). In older adults with age-related vascular changes, this may further compromise oxygen and nutrient delivery, delay tissue repair, and reduce skin tolerance to irritation, contributing to adverse skin outcomes, including pressure injuries and incontinence-associated dermatitis (Wang et al., 2024; McEvoy et al., 2022; Alderden et al., 2025).

We found almost half of the participants used laxatives, with lactulose being the most common. Lactulose increases intestinal osmotic pressure, leading to softer stools and increased defecation frequency, which may contribute to diarrhea and incontinence among older adults with limited mobility or altered consciousness. Repeated exposure to loose stool increases skin moisture, alkalinity, and exposure to fecal enzymes, including proteases and lipases, thereby disrupting the skin barrier and increasing the risk of irritation and inflammation (Gray and Giuliano, 2018; Banharak et al., 2021). In addition, almost all of the participants received antibiotics, with most receiving multiple agents. Antibiotic use may alter the gut microbiota and cause antibiotic-associated diarrhea, increasing stool frequency and prolonging skin exposure to fecal moisture. Prolonged moisture exposure reduces skin tolerance and increases the risk of incontinence-associated dermatitis (Deprez et al., 2023).

Assessment of incontinence-associated dermatitis risk among older adults indicated improvements in both groups following the intervention; however, these findings differed from those reported by Sommana et al. (2024). This discrepancy may be related to differences in intervention duration. The shorter intervention period in the previous study may have limited the opportunity to identify and manage contributing factors, particularly diarrhea. In contrast, the longer implementation period in the present study enabled comprehensive assessment and management of diarrhea through stool examination, appropriate treatment adjustments, and medication management (Banharak et al., 2021; Qiao and Banharak, 2023). Effective diarrhea management can reduce bowel movement frequency, minimize exposure to urine and feces, and decrease the need for frequent changes to absorbent products, thereby improving skin protection and reducing the risk of incontinence-associated dermatitis among older adults.

The incontinence-associated dermatitis care program effectively reduced the severity of dermatitis among older adults, consistent with Gunasegaran et al. (2023). The beneficial effects may be attributed to herbal-based skin protection products containing Centella asiatica and Aloe vera, which promote epithelial repair and proliferation while providing anti-inflammatory, antibacterial, and antifungal effects that support skin healing (Tangjitjareonkun and Supabphol, 2015; Diniz et al., 2023; Su et al., 2025). In addition, these products create a protective barrier that reduces transepidermal water loss and minimizes exposure to irritants from urine and feces, thereby improving skin barrier function and accelerating recovery from incontinence-associated dermatitis.

Comparison of skin pH between groups showed no statistically significant difference at baseline, indicating comparable initial skin conditions. After the intervention, however, a statistically significant difference was observed, demonstrating the effectiveness of the nursing care program in preventing incontinence-associated dermatitis in older adults. The program incorporated evidence-based skin care practices that help maintain normal skin pH, while Centella asiatica and Aloe vera promote cell proliferation and exhibit antimicrobial activity against pathogenic bacteria and fungi (Banharak et al., 2021; Sommana et al., 2024). Together, these effects reduce the risk of secondary infections and promote skin recovery in older adults (Proksch, 2018; Bylka et al., 2014; Liang et al., 2021).

Comparison of skin moisture between groups showed no statistically significant difference at baseline, indicating comparable initial skin conditions. After the intervention, however, a statistically significant difference in skin moisture was observed, demonstrating the effectiveness of the nursing care program in maintaining and improving skin hydration. These findings are consistent with Sommana et al. (2024), who reported that a nursing care program for the prevention and management of incontinence-associated dermatitis helps maintain appropriate skin moisture. This effect may be attributed to evidence-based skin care practices, including gentle cleansing, minimizing skin friction, reducing transepidermal water loss, and providing adequate nutritional support, particularly protein intake, to preserve skin structure, barrier function, and tissue repair (Langer et al., 2024). Collectively, these findings suggest that the structured nursing care program, combined with herbal-based skin protection using Centella asiatica and Aloe vera, effectively protected the skin and maintains optimal skin moisture in older adults with incontinence-associated dermatitis.

## 12 Limitations

This study has several limitations. First, it was conducted in a single semi-intensive care unit within a tertiary hospital in Thailand, which may limit the generalizability of the findings to other clinical settings, such as intensive care units, general medical wards, or healthcare systems in other countries. Second, the intervention was implemented for only 7 days, and no long-term follow-up was conducted after patient transfer or hospital discharge. Third, the relatively small sample size may limit the strength and generalizability of the findings. Finally, the nursing care program was specifically developed for a semi-intensive medical care setting and may require adaptation before implementation in other clinical environments with different patient characteristics.

## 13 Conclusion

We demonstrated that an eight-component nursing care program, combined with an herbal-based skin protectant, was effective in preventing and managing incontinence-associated dermatitis among older adults admitted to semi-intensive and intensive care units. The intervention reduced both the risk and severity of incontinence-associated dermatitis and promoted recovery from the condition. In addition, it helped maintain skin pH within a mildly acidic range, strengthened the skin barrier against irritants, and improved skin moisture.

## 14 Recommendation

In consideration of the limitations of this study, several recommendations are proposed for future research and clinical practice. First, future researchers should expand the settings to include different types of units, hospitals at various levels, or multiple-site studies to increase sample diversity and enhance the generalizability of the findings. Second, long-term follow-up studies are recommended after completion of the nursing care program, such as follow-up after patient transfer to other units or discharge home, to evaluate the sustainability of outcomes and long-term effectiveness of the intervention. Third, future researchers should increase the sample size and include other patient populations beyond older adults to assess the program's applicability and effectiveness across different groups. Finally, the nursing care program for preventing incontinence-associated dermatitis should be further refined and adapted to various clinical contexts. This recommendation includes evaluating its feasibility in real-world settings, acceptance among healthcare providers, and its impact on nursing quality outcomes, to support sustainable implementation in practice.

## Funding

This study was financially supported by Gerontological Nursing Research Cluster, Faculty of Nursing, Khon Kaen University (Grant Number GNRC69-002). Appreciation is extended to this research fund for making this study possible.

## CRediT authorship contribution statement

**Somphorn Chaisomsee:** Writing – original draft, Visualization, Validation, Resources, Project administration, Methodology, Investigation, Funding acquisition, Formal analysis, Data curation, Conceptualization. **Samoraphop Banharak:** Writing – review & editing, Writing – original draft, Visualization, Validation, Supervision, Software, Resources, Project administration, Methodology, Investigation, Funding acquisition, Formal analysis, Data curation, Conceptualization. **Chakkarin Sommana:** Writing – original draft, Visualization, Validation, Software, Resources, Project administration, Methodology, Investigation, Formal analysis, Data curation, Conceptualization. **Sarunya Tuntiyasawasdikul:** Visualization, Validation, Resources, Methodology, Investigation, Formal analysis, Data curation, Conceptualization. **Supin Sim-im:** Visualization, Validation, Project administration, Methodology, Investigation, Formal analysis, Data curation, Conceptualization. **Khanisorn Ransinyo:** Visualization, Validation, Resources, Methodology, Investigation, Formal analysis, Data curation, Conceptualization. **Panita Limpawattana:** Validation, Methodology, Investigation, Formal analysis, Data curation, Conceptualization. **Waraporn Deesui:** Validation, Resources, Methodology, Investigation, Formal analysis, Data curation, Conceptualization. **Porntip Pimpun:** Validation, Software, Methodology, Investigation, Formal analysis, Data curation, Conceptualization. **Teerawat Somkamsri:** Validation, Methodology, Investigation, Formal analysis, Data curation, Conceptualization. **Orada Seeharach:** Validation, Methodology, Investigation, Formal analysis, Data curation, Conceptualization.

## Declaration of competing interest

The authors declare that there is no conflict of interest regarding the publication of this study.
